# Case Report: Two Case Reports of Acute Myopericarditis After mRNA COVID-19 Vaccine

**DOI:** 10.3389/fcvm.2022.827237

**Published:** 2022-03-07

**Authors:** Carlotta Sciaccaluga, Flavio D'Ascenzi, Matteo Cameli, Maddalena Gallotta, Daniele Menci, Giovanni Antonelli, Benedetta Banchi, Veronica Mochi, Serafina Valente, Marta Focardi

**Affiliations:** ^1^Division of Cardiology, Department of Medical Biotechnologies, University of Siena, Siena, Italy; ^2^Unit of Diagnostic Imaging, University Hospital Santa Maria alle Scotte, Siena, Italy

**Keywords:** case report, myocarditis, myopericarditis, mRNA vaccine, COVID-19

## Abstract

**Background:**

Cases of myocarditis and myopericarditis after mRNA COVID-19 vaccines have been reported, especially after the second dose and in young males. Their course is generally benign, with symptoms onset after 24–72 h from the dose.

**Case Summary:**

We report two cases of myopericarditis after the second dose of the mRNA-1273 COVID-19 vaccine in two young males. Both the patients were administered the mRNA-1273 COVID-19 vaccine from the same batch on the same day and experienced fever on the same day of the vaccine, and symptoms consisted of myopericarditis 3 days after the dose.

**Discussion:**

Myopericarditis is usually considered an uncommon adverse reaction after various vaccinations, reported also after the mRNA COVID-19 vaccine. Several explanations have been proposed, including an abnormal activation of the immune system leading to a pro-inflammatory cascade responsible for myocarditis development. Both patients experienced the same temporal onset as well as the same symptoms, it is also useful to underscore that both vaccines belonged to the same batch of vaccines. However, despite these cases, vaccination against COVID-19 far outweighs the risk linked to COVID-19 infection and remains the best option to overcome this disease.

## Introduction

The safety profile of mRNA vaccines for the prevention of COVID-19 disease has been demonstrated in several trials (1–3). Systemic reactions, generally mild and transient, are described mainly after the second dose of vaccine and especially in young people. Few cases of myocarditis post-mRNA vaccine (both BNT162b2 and mRNA-1273 vaccines) have also been reported (4, 5), typically with a benign course. In most case series, symptoms arose 24–72 h after the second dose, whereas only rare cases occurred after 1-week post-vaccine (4).

## Case Description

**Figure 1:**
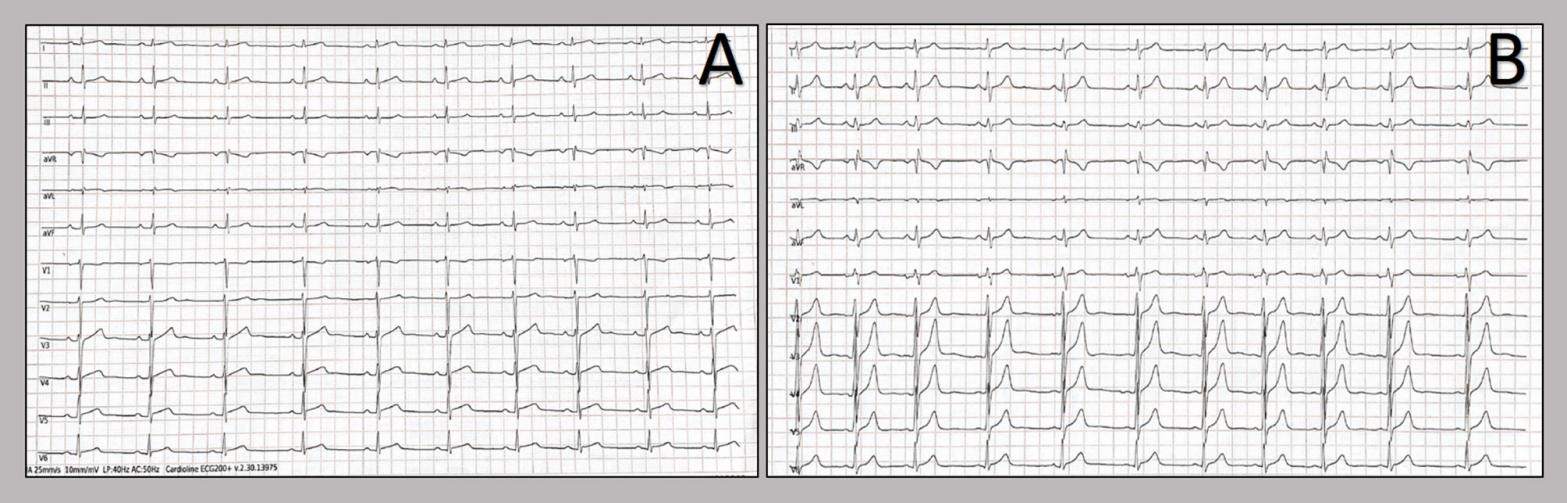
Twelve-lead resting electrocardiograms collected at the hospital admission. Patients' electrocardiograms on admission: 20-year-old patient **(A)** and 21-year-old patient **(B)**. The resting ECGs showed sinus rhythm, normal atrioventricular conduction, incomplete right bundle branch block and no ventricular repolarization abnormalities.

**Figure 2:**
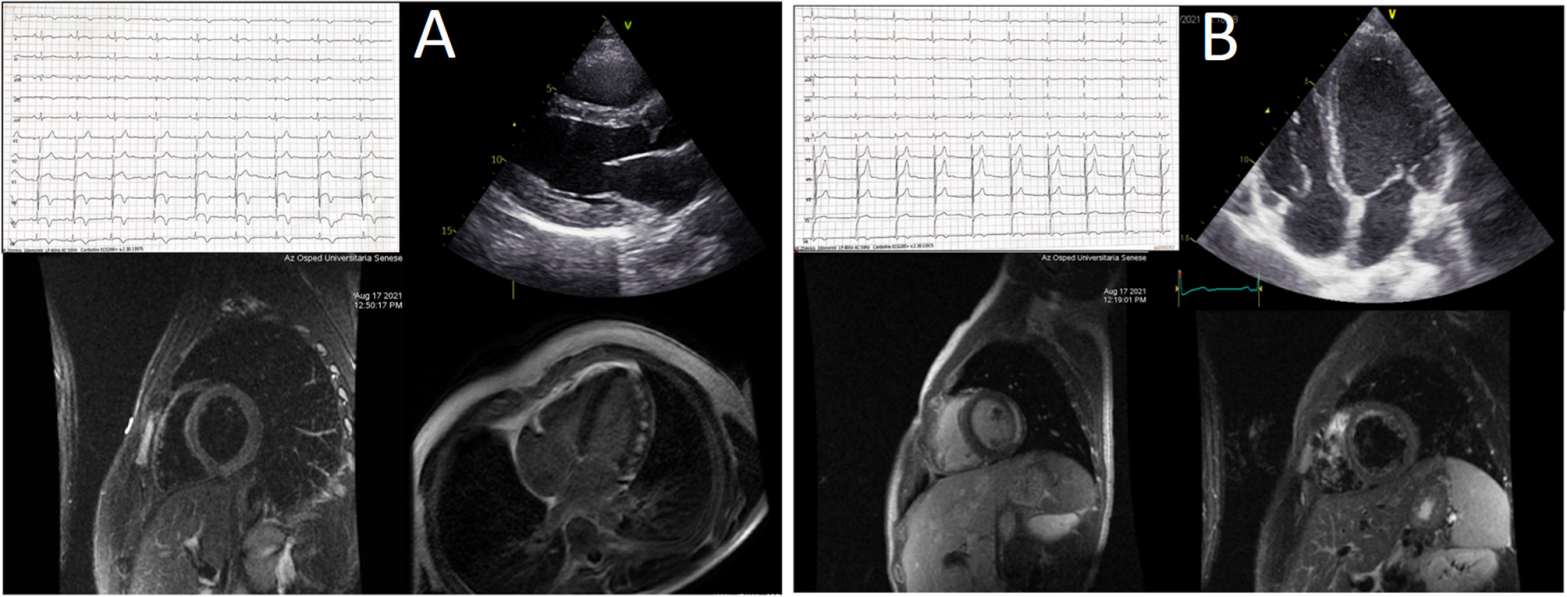
**(A,B)** Central illustration. The picture summarizes the main non-invasive findings in the two patients experiencing acute myopericarditis after mRNA-1273 COVID-19 vaccine. See text for details.

**Figure 3:**
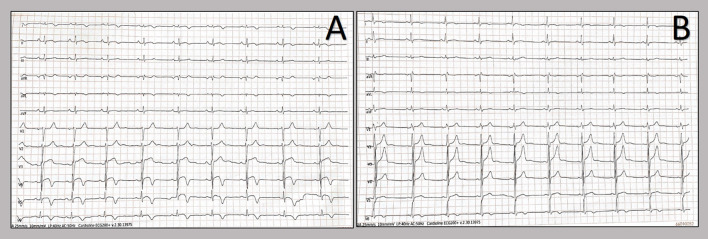
T-wave inversion showed by twelve-lead resting electrocardiograms. Patients' electrocardiograms on day 2 from admission show T-wave inversion in the anterolateral leads in the 20-years old patient **(A)**, whereas in the other patient, the T-wave inversion occurred in the lateral leads **(B)**.

**Figure 4:**
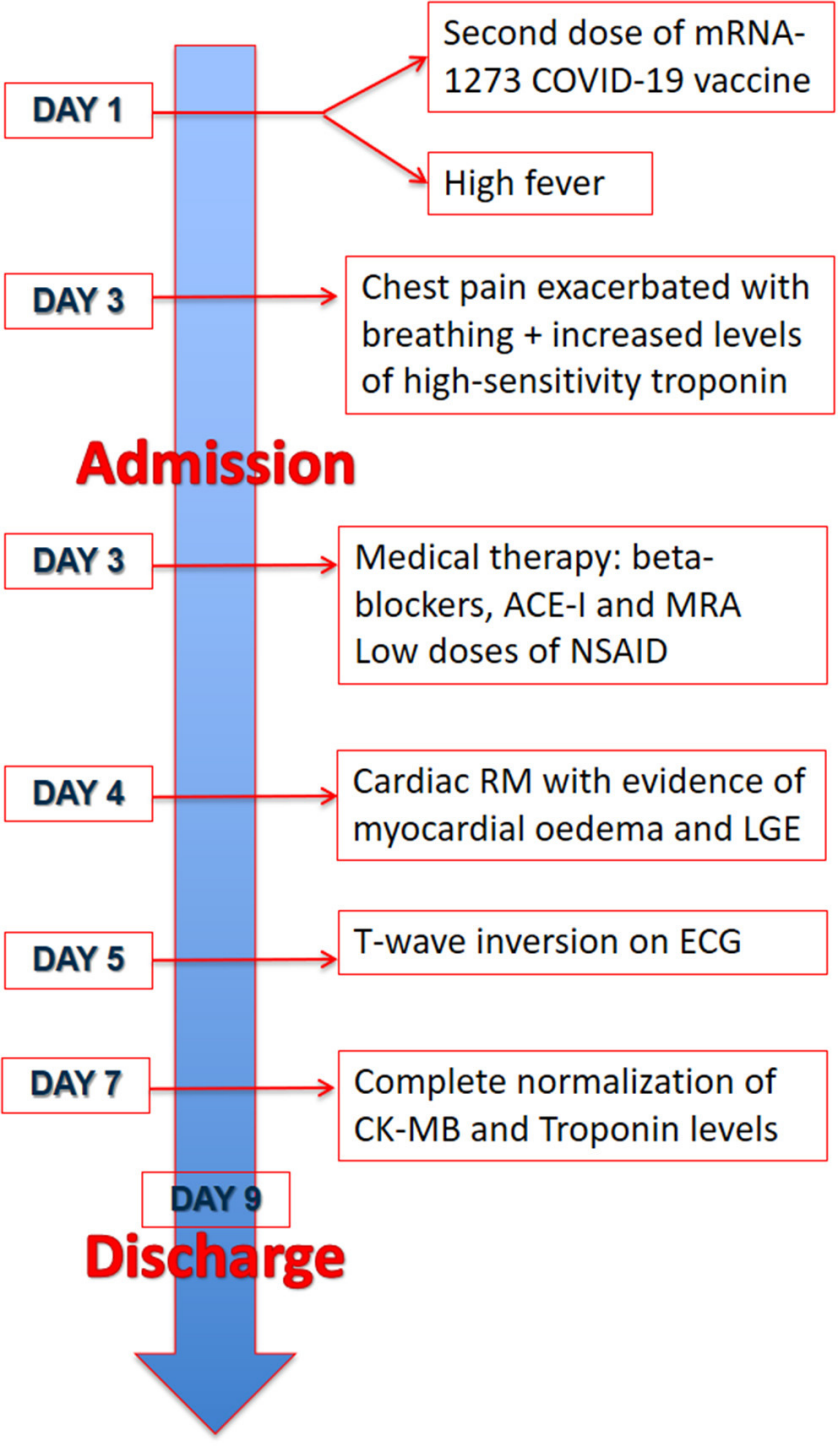
Timeline. Timeline of the two patients from the day of the vaccine and symptoms onset to discharge.

**Figure 5:**
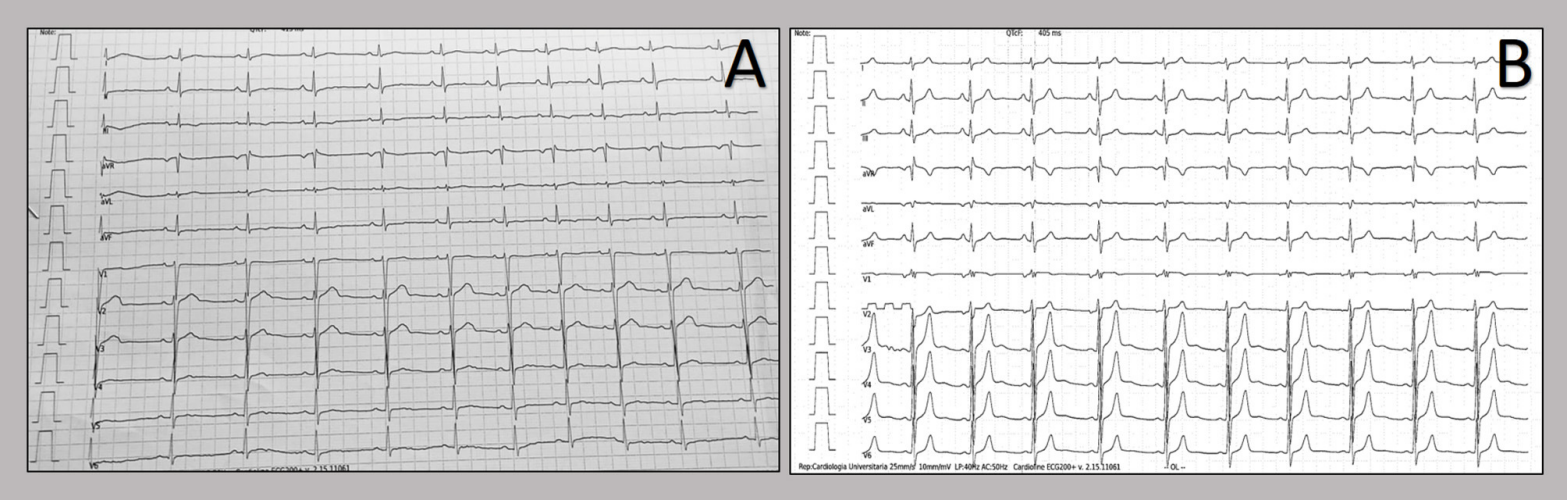
Resting electrocardiograms after hospital discharge. Patients' electrocardiograms after one month from hospital discharge show almost complete resolution of repolarization abnormalities in both patients [in the 20-years old patient **(A)** and in the 21-year old patient **(B)**].

We report two cases of myopericarditis after the second dose of the mRNA-1273 COVID-19 vaccine, from the same batch of vaccines, administered on the same day. Two young males, 20-years old and 21-years old, with no past medical history, experienced fever (38 and 40°C, respectively) on the same day of the second dose of mRNA-1273 COVID-19 vaccine and chest pain, exacerbated with breathing, 3 days later, for which they were admitted to the emergency department. On admission, both patients had normal vital signs with no fever. Nasopharyngeal SARS-CoV2 polymerase chain reaction was negative in both patients. The 12-lead resting electrocardiograms on arrival showed sinus rhythm, normal atrioventricular conduction, incomplete right bundle branch block and no ventricular repolarization abnormalities (Figure 1). Both chest x-rays revealed no significant findings. Blood tests revealed a C-reactive protein = 1.9 mg/dL in both cases, with white blood count within normal limits and increased levels of high-sensitivity troponin (211 and 366 ng/L, respectively). Due to the clinical presentation and the elevation of high-sensitivity troponin, a complete transthoracic echocardiographic exam was performed. In the first case, transthoracic echocardiography showed no pericardial effusion, a mild inferolateral wall thickness (12 mm) with normal biventricular function, absence of wall motion abnormalities and no significant heart valve disease. The echocardiographic examination of the 21-year-old patient showed a minimal pericardial effusion (2 mm) with hyperreflective pericardial layers, normal biventricular function and no significant heart valve disease. Due to the temporal correlation between the symptom onset and the second dose vaccine, the hypothesized diagnosis was acute myopericarditis as an adverse reaction to the mRNA-1273 COVID-19 vaccine. Therefore, on the day after hospital admission, both patients underwent cardiac magnetic resonance (CMR), which confirmed the diagnosis of acute myopericarditis, with evidence of myocardial oedema and late gadolinium enhancement (LGE) with subepicardial pattern (Figure 2A). In particular, in the 20-year old patient, myocardial oedema was found in the middle inferolateral wall, whereas LGE involved the subepicardial region of the lateral wall, inferior basal wall, and anterior apical septum, with left ventricular ejection fraction (LVEF) = 55%. The CMR of the 21-year-old patient revealed myocardial oedema in the mid-basal lateral wall and LGE in the subepicardial region of the basal inferolateral wall and mid-basal lateral wall with LVEF 52% (Figure 2B). The disease course was benign in both patients, and only one patient presented rare ventricular arrhythmias on the admission day (isolated ventricular ectopic beats, 3 couplets and 1 triplet). Due to the patients' low-risk profile and the clear etiology of the myocarditis, we decided not to perform an endomyocardial biopsy. Both patients were treated with low doses of beta-blockers, angiotensin-converting enzyme, and antagonists of mineralocorticoid receptors. Non-steroidal anti-inflammatory drugs were introduced to control chest pain, whereas colchicine was not introduced due to the prevalent myocardial involvement. Serial electrocardiograms showed T-wave inversion in the anterolateral leads in the 20-years old patient, whereas in the other patient, the T-wave inversion occurred in the lateral leads, both occurred 2 days later from admission (Figures 3A,B). Blood tests revealed an initial increase in markers of myocardial injury (peak high-sensitivity troponin 2,474 and 1,414 ng/L and isozyme creatin-kinase MB 80.4 and 50 ng/ml respectively) and C-reactive protein levels (peak 1.9 and 2.16 mg/dl respectively), with a decreasing trend until complete normalization before the hospital discharge. They were both discharged on the 9th day of the in-hospital stay. Figure 4 shows the timeline of these two patients from the day of the vaccine and symptoms onset to discharge. One month after hospital discharge, both patients were asymptomatic and were evaluated by clinical examination, resting ECG and echocardiograms which were all within normal limits. In particular, resting ECG showed almost complete resolutions of repolarization abnormalities (Figure 5).

Their 48-h Holter ECG did not show any brady- or tachyarritmias as well as ST-T dynamic changes. CMR was scheduled at 3 months from the acute event.

## Discussion

Myopericarditis is usually considered an uncommon adverse reaction after various vaccinations (5–7), and few cases have also been described after the mRNA COVID-19 vaccine, both after BNT162b2 and mRNA-1273 COVID-19 vaccines (4, 8). Both vaccines encode the stabilized prefusion spike glycoprotein of SARS-CoV2, and they were recommended as a 2-dose schedule. In certain individuals with genetic predisposition, nucleoside modifications of mRNA might trigger the immune system and the abnormal activation of both innate and acquired immune response (9), leading to a pro-inflammatory cascade responsible for myocarditis development. Besides mRNA immune reactivity, antibodies cross-reaction between SARS-CoV2 spike glycoproteins and myocardial proteins might play a role in post-vaccine myocarditis (10). Furthermore, both age and sex could be factors involved in the development of this adverse reaction (10). In fact, according to several case reports (11) and the large retrospective analysis conducted in Israel (12), the incidence of myocarditis after BNT162b2 mRNA vaccine is significantly higher in young male subjects, hinting that hormonal differences might play a central role in modulating the immune response. Within the Vaccine Adverse Event Reporting System (VAERS), 1,226 reports of myocarditis after mRNA vaccination during the first 6 months of 2021 (13). Furthermore, it has been widely demonstrated that the rate of adverse reactions to the vaccine is significantly lower compared to the rate of complications related to SARS-CoV2 infection (14), also in young individuals (15). In particular, Barda et al. showed that the risk of developing myocarditis after mRNA vaccine is much lower than after SARS-CoV2 infection (2.7 events vs. 11.0 events per 100,000 persons respectively) (16). In line with these results, the Italian Society of Cardiology still recommends vaccination against COVID-19 even in patients that developed myopericarditis after mRNA vaccine (17). However, it recognizes that these patients represent a vulnerable population and therefore some precautions might be taken such as prolonging the interval between the two doses and perhaps choosing a different vaccine for the second dose (17). The two cases we presented showed clinical characteristics in line with the other documented cases and the latest report by Rosner et al. (18): prevalence of male sex, symptoms onset 48–72 h after the second dose of vaccine, and uncomplicated course with mild symptoms. Indeed, both patients, of approximately the same age, were administered mRNA-1273 COVID-19 vaccine on the same day and in the same hospital, and both experienced fever on the same day and symptoms consisted of myopericarditis 3 days after the dose. Furthermore, it is useful to underscore that both vaccines belonged to the same batch of vaccines, questioning whether problems in vaccines storage may be at least in part responsible for these adverse reactions. To the best of our knowledge there are no clear reports linking a storage problem with the onset of systemic adverse reactions, either for COVID-19 vaccine or for other anti-viral vaccines. However, it is well-known that mRNA vaccines require specific handling which might be particularly challenging such as the need to guarantee a correct temperature (19). We actually cannot know whether there had been any problems with the transportation, storage or administration of this batch of vaccine and whether this might have had affected the onset of myocarditis, but it is important to stress this aspect for raising awareness of a possible correlation between these problems and the onset of side effects, which might therefore be explained with a possible toxic effect rather than an immunological pathophysiology.

## Conclusions

We reported two cases of acute myopericarditis in two young males who developed chest pain three days after the second dose of the mRNA-1273 COVID-19 vaccine. Due to several cases of myocarditis after mRNA COVID-19 vaccination, clinical suspicion should be high, especially in young males. However, despite these cases, vaccination against COVID-19 far outweighs the risk linked to COVID-19 infection and remains the best option to overcome this disease.

## Data Availability Statement

The original contributions presented in the study are included in the article/supplementary material, further inquiries can be directed to the corresponding author/s.

## Author Contributions

CS, DM, VM, and BB collected the data upon which the manuscript was based. CS, FD'A, GA, and MG wrote the manuscript, while MC, SV, and MF critically revised it. All authors contributed to the article and approved the submitted version.

## Conflict of Interest

The authors declare that the research was conducted in the absence of any commercial or financial relationships that could be construed as a potential conflict of interest.

## Publisher's Note

All claims expressed in this article are solely those of the authors and do not necessarily represent those of their affiliated organizations, or those of the publisher, the editors and the reviewers. Any product that may be evaluated in this article, or claim that may be made by its manufacturer, is not guaranteed or endorsed by the publisher.
